# Biodegradable Polymer Composites for Electrophysiological Signal Sensing

**DOI:** 10.3390/polym14142875

**Published:** 2022-07-15

**Authors:** Dong Hyun Lee, Taehyun Park, Hocheon Yoo

**Affiliations:** 1 Department of Electronic Engineering, Gachon University, 1342 Seongnam-daero, Seongnam 13120, Korea; danny99hodam@gachon.ac.kr; 2 Department of Chemical and Biological Engineering, Gachon University, 1342 Seongnam-daero, Seongnam 13120, Korea; thpark@gachon.ac.kr

**Keywords:** biodegradability, conductive polymer, frame, adhesive, electrocardiography, electromyography, electroencephalography

## Abstract

Electrophysiological signals are collected to characterize human health and applied in various fields, such as medicine, engineering, and pharmaceuticals. Studies of electrophysiological signals have focused on accurate signal acquisition, real-time monitoring, and signal interpretation. Furthermore, the development of electronic devices consisting of biodegradable and biocompatible materials has been attracting attention over the last decade. In this regard, this review presents a timely overview of electrophysiological signals collected with biodegradable polymer electrodes. Candidate polymers that can constitute biodegradable polymer electrodes are systemically classified by their essential properties for collecting electrophysiological signals. Moreover, electrophysiological signals, such as electrocardiograms, electromyograms, and electroencephalograms subdivided with human organs, are discussed. In addition, the evaluation of the biodegradability of various electrodes with an electrophysiology signal collection purpose is comprehensively revisited.

## 1. Introduction

The long-term and continuous monitoring of human bio-signals such as electrocardiography (ECG) [1,2,3], electromyography (EMG) [4,5,6], electroencephalography (EEG) [7,8,9], and electrooculography (EOG) [10,11] enables early medical diagnosis and recovery from disease. Thus, technologies to accurately monitor human bio-signals are required to reduce the severity of disease [12,13,14]. These electrophysiological measurements record behaviors, including the rhythm and electrical activity in the human brain, heart, and muscles, through the detection of ionic currents flowing in the body [15].

Concerning electrophysiological technology, the most emphasized technical element is the skin-contact electrode, which determines the quality of the bio-signal detection: noise level, signal-to-noise ratio (SNR), and operational stability [16,17]. Skin-contact electrodes are classified into wet-type electrodes [18,19,20] and dry-type electrodes [21,22,23]. The conventional and commercial electrode type is the wet-type electrode, and as a representative example, pre-gelled Ag/AgCl wet-type skin-contact electrodes are extensively used [24,25]. However, the above-mentioned wet-type electrodes suffer from a disadvantage, in that the measurement time is short. The wettability of the wet-type electrodes varies as a function of time, and their conductivity and adhesion to the skin are reduced, which significantly disturbs continuous and long-term detection from an operational stability perspective. Meanwhile, dry-type skin-contact electrodes avoid the issue of a short measurement period. A representative example of a dry-type skin-contact electrode is metal nanofilms, having excellent conductivity and measurement stability [26,27]. However, metal nanofilms still have the limitations of low skin adhesion ability and vulnerability to mechanical stress. As a third alternative, polymer composite-based skin-contact electrodes are emerging [28,29]. Polymer composites provide simple-to-manufacture synthesis with high conductivity, and also have a high mechanical stability. Furthermore, polymer composite-based electrodes offer favorable biocompatibility [30,31] and biodegradability [32,33], which have the advantages that the materials are not harmful to the human body and of being easily disposed of after being used. Biocompatibility and biodegradability, without introducing hazardous substances, allow the electrophysiological devices to have widespread potential applications as wearables with the human body, at any time in daily life.

In this context, we present a focused review of the various polymer composite approaches, concerning the material combinations and their application to versatile electrophysiological sensing. This review provides a comprehensive summary of the biodegradable polymers and their composites, with an emphasis on their biodegradability principles. Furthermore, focused discussions are provided on representative examples of polymer composites-based skin-contact electrodes. Examples of demonstrations of biodegradability using various polymer composites also are given, for use in electrophysiological sensor applications.

## 2. Materials for Biodegradable Polymeric Composites

### 2.1. Overview of Functional Organic Components

Two major approaches have been attempted as a method for imparting biodegradability to epidermal sensors. The first is to construct the entire device from fully biodegradable materials. Substances based on this approach have the advantage that they can be completely decomposed, reducing electronic waste (e-waste) and allowing recycling. These materials are also suitable for use as implantable devices in the body. However, to realize a material that is easy to degrade and that has excellent mechano-electrical properties at the same time, careful design of the material is required. The second method is blending functional organic components with different benefits. This approach allows imparting the desired functionality to composite electrodes. Most devices belonging to this type are composed of a biodegradable matrix and a conductive material. Due to these structural compositions, composite electrodes exhibit partial degradation. The physical characteristics of polymer composites mentioned above vary depending on which functional materials are blended. In the case of biodegradable polymer electrodes, functional organic components are classified into substances that provide conductivity, mechanical stability, and skin adhesion, respectively. This chapter introduces each functional substance and the mechanisms that impart various properties.

### 2.2. Conductive Polymeric Materials

Conducting polymeric materials (CPMs) have been highlighted in various research fields, such as in photovoltaic cells [34,35], displays [36,37], chemical sensors [38,39], optical detectors [40,41], and energy storage systems [42,43], due to their adjustable ion and electronic conductivity, optical transparency, and low-temperature process suitability. In addition to the mentioned applications, CPMs can be utilized as a key component for epidermal organic electrodes in the following manners: First, due to the excellent compatibility between CPMs and common organics, polymer composites with desired properties can be easily implemented. Second, unlike metal-based conductors, the compatible CPMs are free from issues such as radioactivity or toxicity.

**Figure 1:**
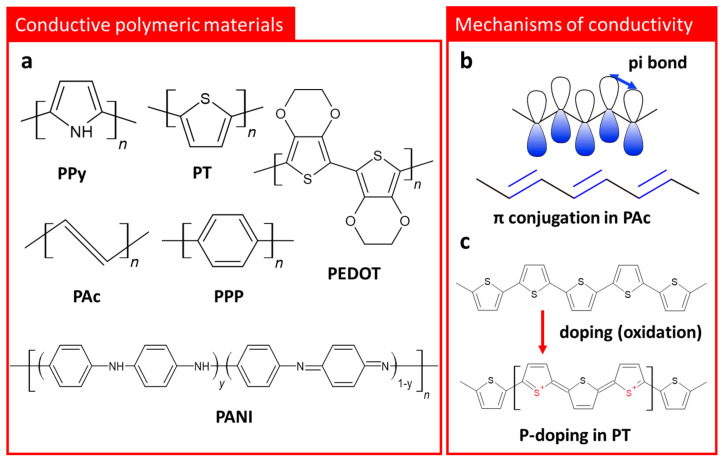
(**a**) Chemical formulas of various conducting polymeric materials: PPy, PT, PAc, PPP, PEDOT, and PANI. Schematic description of (**b**) conjugated π -bonds in PAc, and (**c**) p-doping mechanism in PT chain through oxidation.

Since polyacetylene (PAc) was first reported as a conductive polymer [44], in 1977, various CPMs such as polypyrrole (PPy) [45], polythiophene (PT) [46], poly(p-phenylene) (PPP) [47], poly(3,4-ethlyenedioxythiophene) (PEDOT) [48], and polyaniline (PANI) [49] have been developed (Figure 1a) and studied regarding their physical and chemical characteristics. As shown in Figure 1a,b, CPMs have a Pi (π)-conjugated system, consisting of irregular single and double bonds along the polymer chain [50], in their molecular structures. The conductivity of CPMs originates from the above-mentioned conjugated carbon chains containing highly delocalized, polarized, and electron-dense π bonds [50,51]. To reach a conductivity similar to metallic conductors, CPMs have typically needed to be doped or ordered into a high crystalline phase. Oxidation or reduction of CPMs for negative or positive polarons/bipolarons in the polymer chain are utilized. For instance, the removal of π-bond electrons from the conjugated backbone generates radical cation defects (known as polarons) and delocalizes the remaining electrons in the π -orbitals, which allows the electrons to move freely along the chain. The formation of a positively charged polymer backbone involves the incorporation of dopant anions, which have the effect of maintaining the electrostatic balance of the positive charge (Figure 1c). Positively charged structures provide p-doping, and negatively charged structures provide n-doping [52]. The delocalization of these polarons/bipolarons along the polymeric backbone improves the conductivity of CPMs. For instance, PAc generally exhibits a conductivity of 10^−5^ S cm^−1^, but its conductivity can be further improved to the level of 10^2^ to 10^3^ S cm^−1^ through appropriate doping. In the case of PPy, a representative CPMs, it has an extremely low conductivity, similar to insulators in an undoped state, but by adding halogenic dopant materials during the synthetic process, the conductivity of PPy increases drastically to 10^−3^ S cm^−1^ [52]. Poly(3,4-ethylenedioxythiophene) polystyrene sulfonate (PEDOT:PSS), which is the most widely used CPM commercially, is a good example of utilizing the ability to control the conductivity according to doping level. The conductivity of PEDOT:PSS can reach up to 4600 S cm^−1^, and that it has availability for conductivity improvement not only through the synthesis process, but also via post-treatments, is an attractive point.

Although the conductivity of CPMs can be further improved by doping or increasing crystallinity, their natural characteristics, such as brittleness, non-biodegradability, and weak mechanical durability, limit their use as robust skin contact electrodes. As a strategy to overcome the mentioned issues, CPMs have been studied and developed in the form of composites blended with other functional matrices.

### 2.3. Biodegradable Frame Materials

**Figure 2:**
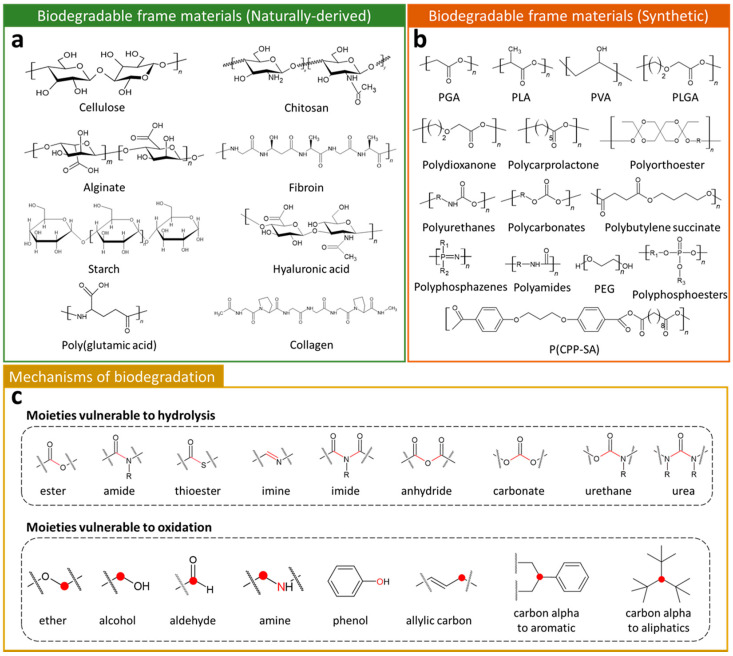
Chemical formulas of (**a**) naturally-derived and (**b**) synthetic biodegradable polymers. (**c**) Chemical structural formulas of functional groups capable of hydrolysis or oxidation reactions. Red line, circle, or character highlight the specific position in moieties.

Biodegradable frame materials (BFMs) are biodegradable matrix components in polymer composites. Their chemical structure, molecular weight, crystal structure, and molecular interaction with CPMs have a significant influence on the mechanical stability, hydrophilicity, hydrophobicity, biocompatibility, and degradation rate of the final composite [53]. As shown in Figure 2a, various naturally-derived polymers, such as cellulose [54], chitosan [55], alginate [56], fibroin [57], starch [58], hyaluronic acid [59], poly(glutamic acid) [60], and collagen [61], have been reported for their biodegradable characteristics. Due to their similarity of macro biomolecules, a lot of naturally-derived BFMs exhibit high biocompatibility for use as implantable temporary components in medical technology [62]. In the case of synthetic BFMs, these possess excellent tunability of the desired chemical and physical characteristics, such as molecular weight, functional groups, or arrangement of monomers. The tailored synthetic BFMs exhibit physiochemical and mechanical properties comparable to biological tissues [63]. Since poly(glycolide) (PGA) was first marketed as a biodegradable synthetic polymer in the 1960s [53], various BFMs, including poly (lactide) (PLA) [64], poly(vinyl alcohol) (PVA) [65], poly(lactide-*co*-glycolide) (PLGA) [66], polydioxanone [67], polycarprolactone [68], polyorthoester [69], polyurethanes [70], polycarbonates [71], poly(butylene succinate) [72], poly(phosphazenes) [73], poly(1,3,-bis-(p-caboxyphenoxy propane)-*co*-(sebacic anhydride)) (P(CPP-SA)) [74], polyamides [75], polyphosphoesters [76], and poly(ethylene glycol) [77], have been developed and reported (Figure 2b).

Biodegradation refers to the phenomenon that occurs through the activity of enzymes or chemical degradation by living organisms and their secretions [78]. During biodegradation, organic polymers are degraded into small molecular weight fragments that can ultimately be further converted into carbon dioxide and water. As mentioned above, the basic degradation mechanism is to divide macro molecular weight polymers into a smaller unit, and most BFMs have specific points where they are easily decomposed by hydrolysis and oxidation (Figure 2c). For instance, polymers containing ester bonds can be hydrolyzed under acidic or alkaline conditions. The specific moieties, such as ester, amide, thioester, imine, imide, anhydride, carbonate, urethane, and urea, are major unstable sites, where hydrolysis reactions occur. Degradation of polymers is also able to progress through oxidation of specific sites (moieties) in the polymer backbone or side chains. The common oxidizable groups, such as ether, amide, aldehyde, amine, phenol, allylic carbon, carbon alpha to aromatic, and carbon alpha to aliphatic, provide biodegradability to both synthesized and naturally-derived BFMs.

### 2.4. Biocompatible Adhesives

**Figure 3:**
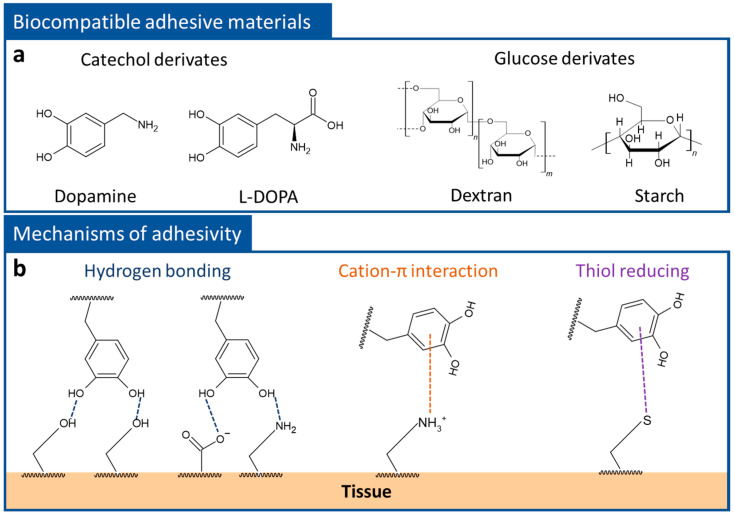
(**a**) Biocompatible adhesive materials are classified into catechol derivates (dopamine, and 3,4-dihydroxy-L-phenylalanine (L-DOPA)) and glucose derivates (dextran and starch). (**b**) Schematic description of the tissue adhesivity of catechol derivates: hydrogen bonding, cation-pi interaction, and thiol reduction.

To obtain clear electrophysiological signals and reduce noise, a stable contact state between electrodes and the skin surface is one of the most important requirements. In general, the surface of the skin is known to be comprised of a protein called keratin [79,80]. Keratin has specific functional groups, such as hydroxyl (-OH), carboxyl (-COOH), thiol (-SH), and amine (-NH_2_) groups [79], and skin adhesion of the polymer composites can be obtained by inducing the interaction between these functional groups on the keratin surface. Among the various functional organic materials, catechol derivatives and glucose derivatives have been highlighted as biocompatible adhesives [81,82,83,84] (Figure 3a). For instance, dopamine, which was inspired by the mussel, exhibits excellent adhesive properties for tissue repair, drug delivery, and skin contact patches, due to its catechol groups [85,86,87,88]. The catechol group consists of two hydroxyl groups and a phenolic group and this unique combination allows it to participate in various interfacial adhesions, such as hydrogen bonding, cation-pi interactions, and thiol reductions [86] (Figure 3b). In the case of glucose derivates, including dextran and starch, these contain a lot of hydroxyl groups in the monomer. The hydroxyl groups have multiple roles, providing bonding between monomers and adhesion to the keratin surface through hydrogen bonding.

## 3. Electrophysiological Signal Sensing

### 3.1. Electrophysiological Signals

**Figure 4:**
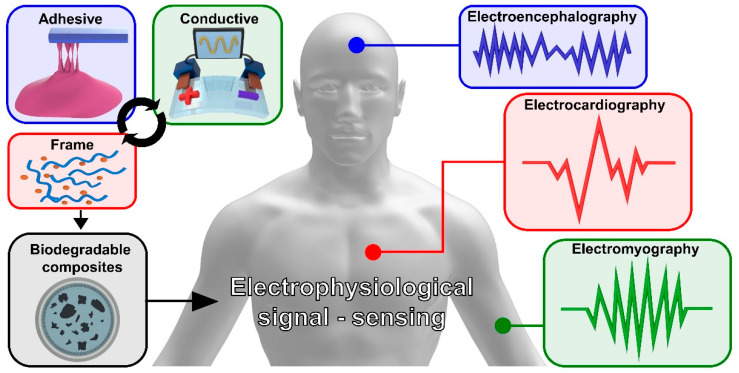
A 3D schematic of human electrophysiological signals and biodegradable composite polymer electrodes.

Electrophysiology is the study of electrical activity, by interpreting recorded electrical changes between cells and tissues. It is possible to diagnose diseases of the human body by recording changes in the electrical activity of core organs, such as the heart, brain, and muscles. The electrical activity extracted from the core organs could be applied for various purposes, including early disease diagnosis [89,90,91], artificial organs [92], and augmented reality [93]. The methods of collecting electrophysiological signals are classified into an invasive method and a non-invasive method. The invasive method has the advantage of being able to accurately collect a signal by implanting electrophysiological sensors into the human body. However, the toxicity of the implanted electrophysiological sensor may cause damage to the human body, and additional surgery is required to remove the non-decomposable electrophysiological sensor at the end of its lifespan. On the other hand, the non-invasive method has the advantage of easily collecting electrophysiological signals and a lower burden of surgery, due to attaching an electrophysiology sensor to the human skin. However, the signal of the targeted human organ is indirectly transmitted through the skin. Therefore, the measured electrophysiological signal is affected by the impedance of human skin. In addition, electrophysiological signals collected by the non-invasive method are sensitive to various external environmental factors, such as humidity, noise, and vibration. As a result, invasive and non-invasive electrophysiological sensors are needed with biocompatible and non-toxicity materials for human health. In addition, electrophysiological sensors based on toxic and non-decomposable substances could cause environmental pollution, due to an increase in the e-waste [94]. As such, the development of biodegradable material-based electro-physiological sensors is highly in demand. In this chapter, we report on biodegradable polymer electrodes for the measurement of electrocardiography (ECG), electrocardiography (EMG), and electroencephalography (EEG) (Figure 4).

### 3.2. Electrocardiography

**Figure 5:**
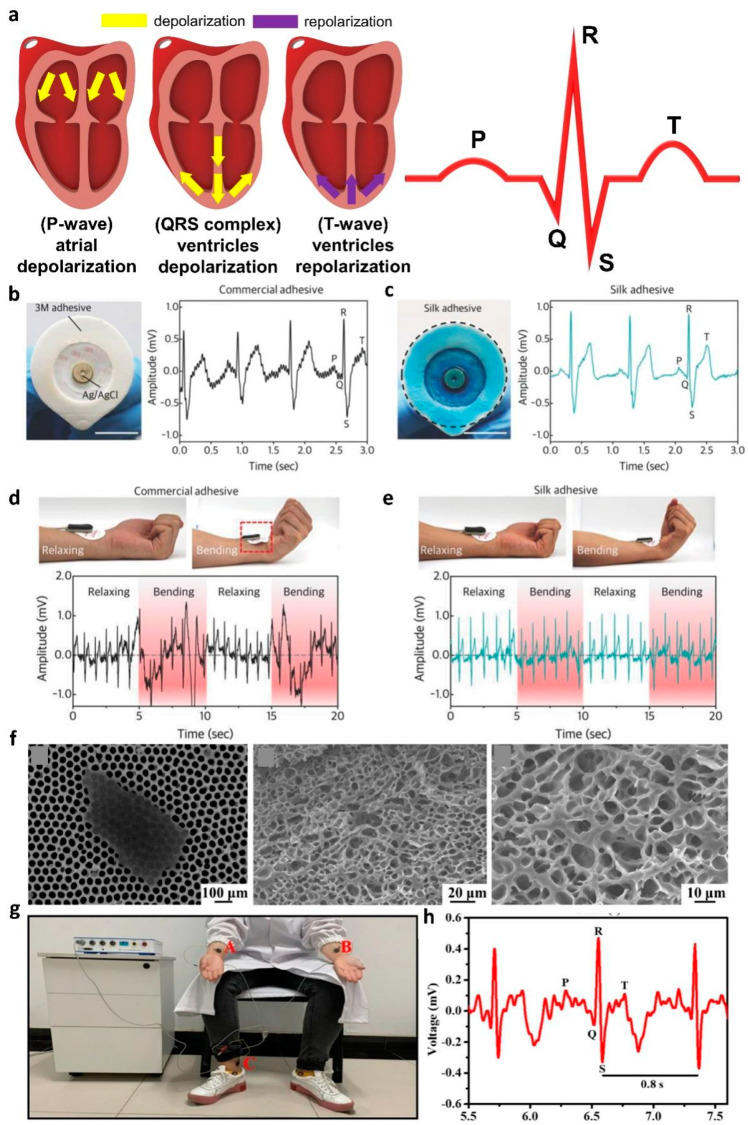
(**a**) The depolarization and repolarization of the heart for generation of a P-wave, QRS complex, and T-wave; (**b**) commercial Ag/AgCl electrodes and collected ECG waveforms; (**c**) silk fibroin adhesive applied electrodes and collected ECG waveforms; (**d**) commercial Ag/AgCl electrodes and collected ECG waveforms with wrist movement; (**e**) silk fibroin adhesive applied electrodes and collected ECG waveforms with wrist movement (adapted from [99] with permission from the John Wiley and Sons); (**f**) SEM images of a delaminated MXene sheet and the freeze-dried MXene-PAA-ACC hydrogel; (**g**) photograph of electrode positions for ECG measurement; (**h**) ECG waveform collected with MXene-PAA-ACC hydrogel (adapted from [100] with permission from the American Chemical Society).

An ECG records the electrical activity produced by the heartbeat. ECG signal analysis is used to confirm a normal heart rate and for early diagnosis of various heart diseases, such as arrhythmias [95], myocardial infarction [96], and angina [97]. The ECG signals generated by a single heartbeat are classified in the following order: P-wave, QRS complex, and T-wave [98]. The P-wave and the QRS complex are generated by the atria depolarization and the depolarization of the ventricles, respectively. Finally, the T-wave occurs with the repolarization of the ventricles (Figure 5a).

In 2018, Seo et al. demonstrated an ECG sensor using calcium (Ca)-modified silk fibroin as an adhesion layer [99]. The reusable Ca-modified silk fibroin adhesive exhibited multifunctionalities, such as conductivity and water-degradability. When immersed in water, the peel strength of Ca-modified silk fibroin adhesive decreased to 1.2 N m^−1^ within 30 min and could be separated from the skin without any damage. To confirm the effect of the adhesive on ECG, they compared and analyzed the ECG waveforms collected by a commercial Ag/AgCl electrode and silk-adhesive-layer-based electrode. The commercial Ag/AgCl electrodes presented PQRST peaks with high noise levels and low signal amplification, due to weak adhesion and relatively high impedance (Figure 5b). In contrast, the electrodes with the Ca-modified silk adhesive presented lower noise levels and higher signal amplification than the Ag/AgCl electrodes, due to their low impedance and high adhesion properties (Figure 5c). In addition, the proposed silk-adhesive ECG electrode exhibited an undistorted ECG signal, despite the movement caused by the bending of the wrist (Figure 5d,e).

To fabricate a multifunctional hydrogel electrophysiology sensor, in 2021, Li et al. demonstrated a conductive hydrogel electrode, by blending MXene, poly(acrylic acid) (PAA), and amorphous calcium carbonate (ACC) to measure ECG signals [100]. In this work, Mxene, PAA, and ACC were used as conductive, biodegradable, and cross-linking components. The Ca ion of ACC interacts with the carboxylic group of PAA and -OH, -F, and -O groups at the surface of Mxene through metal chelation. With the supramolecular interaction between the components, the proposed MXene-PAA-ACC hydrogel exhibited strong self-healable, stretchable, and biodegradable characteristics. The proposed hydrogel electrode degrades within 65 days in phosphate-buffered saline (PBS) solution. The degradability of the MXene-PAA-ACC hydrogel originated from its chemical and structural characteristics. The surface-terminated functional groups of hydrophilic MXene nanosheets and the carboxyl groups of PPA increased the water penetration of MXene-PAA-ACC hydrogels. Furthermore, the hydrogel electrode has porous microstructures, to facilitate the penetration of water molecules in PBS (Figure 5f). Water molecules penetrating the gel swell the MXene-PAA-ACC hydrogels by intercalating between the MXene nanosheets or binding to the hydrophilic PAA. As the cross-linking between components gradually weakens due to swelling, the gel eventually decomposes. For ECG signal measurement, MXene-PAA-ACC hydrogel electrodes were attached to human arms and ankles (Figure 5g). Figure 5h shows the ECG waveform measured with the proposed electrode. The measured ECG exhibited the heart electrical activity of normal adults and distinguishable PQRST peaks.

Yuen et al. collected an ECG signal using polycarbonate (PC)-based ion gels and poly(3,4-ethylenedioxythiophene) polystyrene sulfonate (PEDOT: PSS) electrodes [71]. To achieve robust adhesivity and high conductivity, PCs were prepared using N-substituted cyclic carbonates. The designed PCs were synthesized thorough the ring-opening polymerization of polyethylene glycol (PEG), mono (8MC (6-methyl-1,6,6-dioxazocan-2one)), and bifunctional cyclic carbonates (bis 8MC (6,6′-(ethane-1,2-diyl)bis(1,3,6,-dioxazocan-2-one)). The reaction was initiated by the introduction of the primary alcohol group of PEG with the presence of an organic catalyst. Ionic liquids ([BMIm][Lac], 1-butyl-3-methylimidazolium chloride) were intercalated during the synthetic process to enhance the conductivity of the final gel electrode. The proposed PCs based ion gels are non-toxic and have good biodegradability in water. Owing to the large amounts of ester bonds in the PC chains, the hydrolysis reaction occurred at the ester moieties when gels were submerged in water. The polycarbonate-based ion gels were attached to the volunteer’s forearm and elbow, to collect the ECG using a three-electrode method. The proposed gel electrode exhibited a clearer ECG signal sensing performance than the commercial Ag/AgCl electrode, due to a low impedance level.

### 3.3. Electromyography

**Figure 6:**
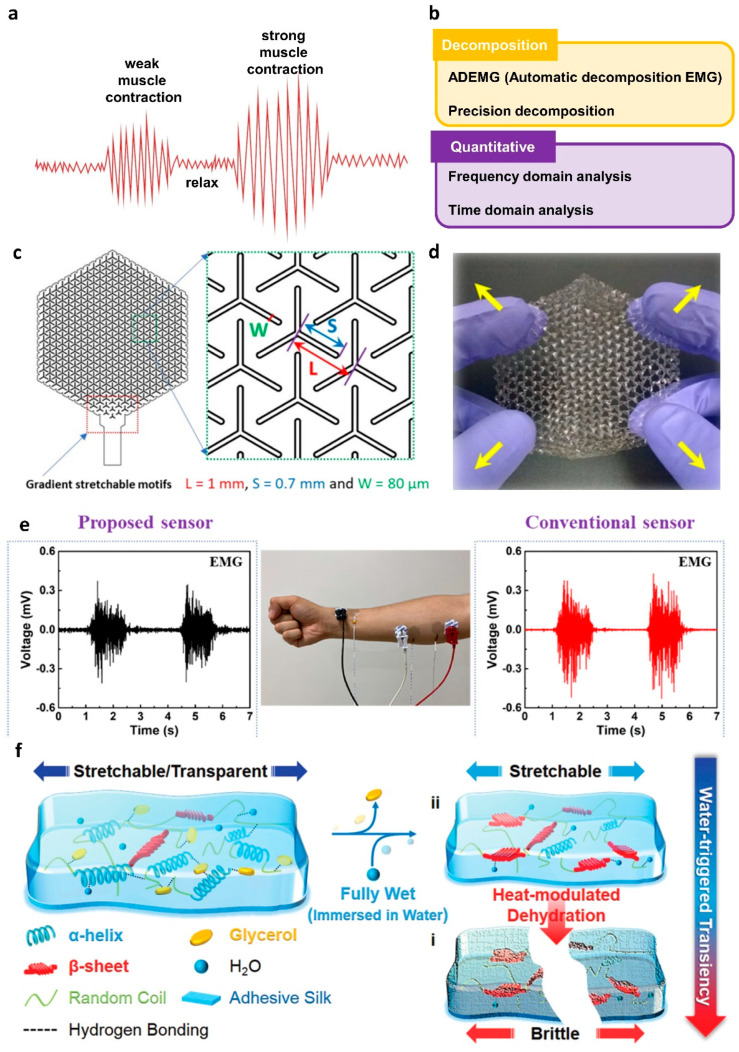
(**a**) Schematic of EMG in muscle contraction and relaxation; (**b**) table of quantitative EMG analysis; (**c**) design of the Y-shaped kirigami electrode; (**d**) photograph of the stretching characteristic of the Y-shaped kirigami electrode (adapted from [109] with permission from American Chemical Society); (**e**) EMG measurements for the proposed electrode and conventional electrode; (**f**) schematic of water-induced changes in GEPC substrate-based TEE electrodes (adapted from [110] with permission from the John Wiley and Sons).

EMG is a method that records the electrical activity derived from the contraction and relaxation of muscles. EMG waveforms are generated by action potentials during muscle contraction and stabilization states. The stronger the contraction of the muscle, the higher the action potential (Figure 6a). However, it is difficult to quantitatively determine the EMG signal because the composition and density of muscle tissue varies from individual to individual and the thickness of the skin is different. To overcome this issue, efforts have been made to decompose and interpret EMG signals (Figure 6b). The automatic decomposition EMG (ADEMG) method [101] and the algorithm of precision decomposition [102] are techniques for decomposing EMG signals. In the case of EMG signal quantification, frequency domain [103] and time domain [104,105] analysis have been used. In general, the collected EMG potentials are used for the diagnosis of muscle diseases. Furthermore, since the muscles are operated by nerves, EMG can be utilized for various neurological diagnoses, such as peripheral neuropathy [106] and trigeminal neuralgia [107].

In 2020, Xu et al. reported a cost-effective and disposable EMG sensor by patterning it on a paper substrate with graphite from a commercial 9B pencil [108]. The paper substrate was composed of porous cellulose, which can be easily dissolved in water through a hydrolysis reaction between monomers. The water-based degradation performance of the proposed device was easily controlled by the coating of Silbione RT Gel 4717 A/B. The additional Silbione coating provided the film with skin adhesion and waterproofing properties. The pencil–paper sensor was adapted to collect EMG signals from the forearm. The proposed EMG sensor exhibited stable and real-time EMG signal sensing performance under direct water injection and sweaty skin conditions.

A method to improve sensing performance by utilizing structural changes was reported. In 2021, Won et al. collected EMG signals by fabricating a sensor consisting of PLA as a BFM and PEDOT: PSS as a CPM [109]. The proposed sensor was laser cut in a Y-shape, to accommodate a kirigami structure with increased stretchability and areal coverage. Figure 6c shows the design of the Y-shaped kirigami structure electrode, adjusting L and S variables to control the stretchable characteristics. Moreover, the W variable controls the area of the actual electrode. With the aforementioned structure, the proposed electrode exhibited stretchability in the diagonal direction (Figure 6d). Figure 6e shows the EMG potentials measured in the vicinity of the forearm flexor with the conventional Ag/AgCl gel electrode and the proposed electrode. A higher SNR value was achieved when using the proposed electrode for EMG measurement, due to an enlarged areal coverage. Owing to the usage of PLA, the proposed device has potential for water-degradability under acid or base conditions through the catalyzed hydrolysis reaction. The ester bonds of PLA can be easily broken down by the water molecules via random chain scission.

To manufacture an eco-friendly electrophysiological sensor that can be easily separated and with strong adhesion to the skin, in 2020, Zhang et al. demonstrated a transient epidermal electronic (TEE) sensor based on a genetically engineered plasticized copolymer (GEPC) for EMG monitoring [110]. The GEPC was prepared through modification of silk fibroin with resilin protein and plasticized with glycerol. In the composite gel, all components were interconnected by hydrogen bonding. The proposed TEE presents step-wise water-degradability, due to the interaction between water and its components (silk fibroin, resilin protein, and glycerol). Figure 6f shows a sequential schematic of the TEE degradation by water. Decomposition occurs when the TEE is immersed in water, but it retains its conductivity and adhesivity until this stage. During this process, the glycerol was extracted from the gel and the plasticizer was exchanged with water molecules. However, after heat-modulated dehydration, the gel lost its stretchable characteristics and easily broke down into small fragments, owing to the absence of a plasticizer.

### 3.4. Electroencephalography

**Figure 7:**
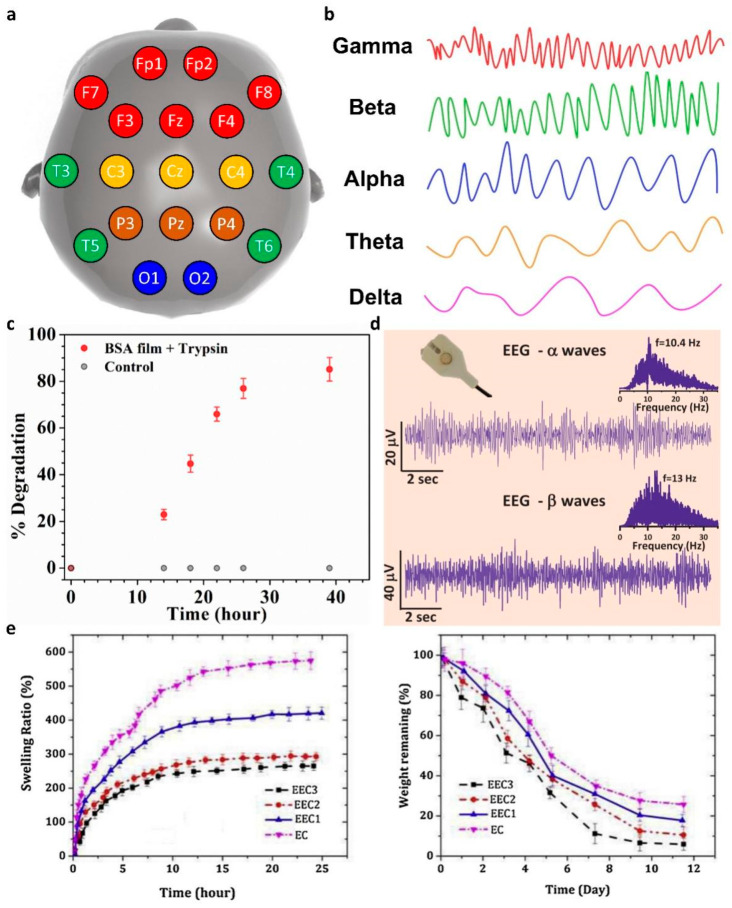
(**a**) Schematic of the international 10–20 EEG electrode system method; (**b**) EEG waveform classified by the power spectral method; (**c**) degrading rate of BSA film in a trypsin condition; (**d**) EEG waveform of alpha and beta waves (adapted from [115] with permission from John Wiley and Sons); (**e**) swelling rate and degradation rate of conduct polymer coatings (adapted from [116] with permission from the Elsevier B.V.).

EEG is a method that records the brain signals caused by the electrical activity of the nervous system. The locations of human scalp electrodes for EEG measurements are classified according to international standards. Figure 7a shows a map of human scalp electrode positions with a 10–20 system method. The electrode was labeled as frontal (F), temporal (T), parietal (P), occipital (O), and central (C), respectively. Additionally, the FTOPC abbreviation was followed by a number, to define the detailed position for the EEG measurement [111,112]. Since an EEG waveform measured in the human brain is visually confusing and difficult to analyze, the complex signal is generally interpreted with the frequency, using the power spectrum method (Figure 7b) [113,114]. First, the delta waves are vibrations below 4 Hz and appear in meditation or coma. The theta waves vibrate at 4~7 Hz and appear in the process of recalling memories or initiating sleep. The alpha wave appears at about 8~12 Hz and is the EEG in a stable state. The Beta waves that are generated in a state of arousal have a frequency of 13~25 Hz. Finally, the gamma waves that occur above 25 Hz are caused by a high wakefulness state and REM sleep.

In 2021, Nandi et al. demonstrated an EEG sensor based on bovine serum albumin (BSA) protein extracted from bovines [115]. The proposed polymer gel composite exhibited high proton-conductivity, owing to the hydrogen bond network between oxo-amino-acids functional groups in the protein and the water molecules. The proton-conducting polymer gel was prepared through the polymerization of reduced BSA (R-BSA). First, BSA was treated with trifluoroethanol, water, and 2-mercaptoethanol for reduction of BSA. After that, the R-BSAs were polymerized using ethylenediamine via reshuffling of the disulfide bonds of R-BSA. The prepared gel composite exhibited a 500% stretchability and a proton conductivity of about 5 mS cm^–1^. Figure 7c shows the protease-based biodegradable characteristics of R-BSA films. Trypsin, which is a BSA digesting enzyme through a hydrolysis reaction, was added to tris-buffer solution (pH 7.4, 37 °C). A R-BSA film treated with trypsin for 40 h was degraded by about 80%. Figure 7d shows the collected EEG signals using a R-BSA-based electrophysiology sensor. Clear alpha waves and beta waves were successfully measured according to the closed and open states of the eyes.

To investigate the electrical activity of the cerebral cortex, in 2016, Yu et al. demonstrated EEG and electrocorticography (ECoG) sensors utilizing silicon nanomembranes (Si NMs) [116]. In the proposed sensor, Si NMs, PLGA, and SiO_2_ were used as electrodes, substrates, and a passivation layer. The PLGA and SiO_2_ dissolved within 4–6 weeks and 6 months under the condition of 37 °C biofluids, respectively. The dissolving mechanism of components including Si NMs, SiO_2_, and PLGA is a hydrolysis reaction. Si and SiO_2_ can form ortho-silicic acid (Si(OH_4_)) under biofluid conditions. In the case of PLGA, the presence of a specific moiety (ester bond) of PLGA provides strong water degradability. The proposed sensor was attached to the cortex of the left hemisphere and the periosteum of an adult rat, to collect EEG and ECoG signals. The measured ECoG signal showed similar waveforms to the signal recorded by the commercial control electrode. In the case of EEG signals, reliable theta waves and sleep spindles were recorded through the proposed electrode based on in vivo sensing.

Meanwhile, Manouchehri et al. designed a conductive coating material to increase the biocompatibility of the electrode–tissue interface [117]. The proposed conductive coatings were prepared by adding oligoaniline to chitosan and epoxidizing the chitosan by reaction with epichlorohydrin. Figure 7e shows the degree of swelling and degradation according to the oligoaniline content of the conductive coatings. When the content of hydrophobic oligoaniline was decreased, the diffusion of water molecules inside the hydrogel was relatively increased and this accelerated the swelling rate. According to the increased diffusion of water molecules, the decomposition rate was promoted. During the swelling process, the cross-linking interaction between the amine group of chitosan and the carboxyl group of the oligoaniline began to be weakened by the water molecules, and as this process continued, the gel broke down into small pieces.

### 3.5. Biodegradability Evaluation of Polymers

Biodegradable polymer-material-based electrophysiological sensors are attracting attention due to the growing importance of environmental protection. The conventional electrophysiological sensors are manufactured based on toxic synthetic materials and non-degradable materials, to improve their functions. However, human health is threatened by the increase in e-wastes due to the high demand for electronic products. Recently, research on electrophysiological sensors using biodegradable polymers has been increasingly reported, to create a green environment. The biodegradability of a polymer is primarily due to the principle of releasing polymer-binding chains through various microorganisms, water, and biofluids. In addition, naturally extractable guar gum [18], gelatin [118], starch [119], and fabric [120,121] have been adopted for achieving green electrophysiological devices, by fabricating biodegradable polymers. Furthermore, research is underway to improve parameters such as the conductivity, biocompatibility, and stability of biodegradable polymers. In this chapter, we report the biodegradation results of electrophysiological sensors fabricated with biodegradable polymers.

**Figure 8:**
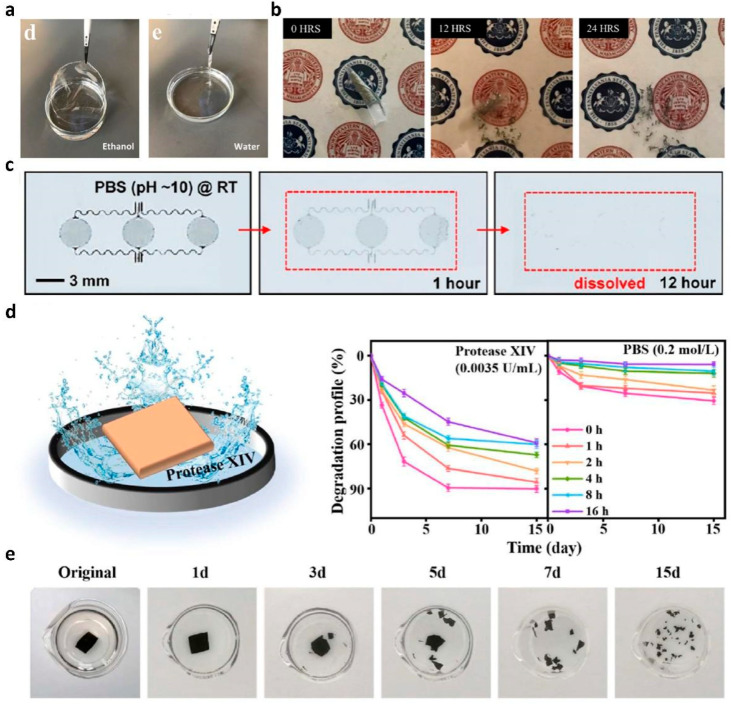
(**a**) Photograph of A Galactomannan film immersed in ethanol and water for 24 h; (**b**) photograph of the proposed Zn electrode immersed in ethanol and distilled water for 24 h (adapted from [122] with permission from the American Chemical Society); (**c**) photographs of the transient electrode immersed in PBS solution (PH 10) at room temperature (adapted from [123] with permission from the American Chemical Society); (**d**) schematic of the Ppy@AM-SF/CNC films immersed in protease XIV solution (left) and the weight loss change of the Ppy@AM-SF/CNC films in protease XIV solution and PBS solution (right); (**e**) photographs of the Ppy@AM-SF/CNC films immersed in protease XIV solution over 15 days (adapted from [124] with permission from the Elsevier B.V.).

To manufacture an eco-friendly biodegradable sensor, in 2018, Yi et al. demonstrated a Zn-based ECG and EMG sensor with strong biodegradability using galactomannan extracted from Leucaena leucocephala (L. leucocephala) as a substrate [122]. Both Zn and galactomannan exhibited a solvent-selective degradation, owing to the hydrolysis reaction. Galactomannan substrates are structurally stable in ethanol but decompose in water (Figure 8a). In addition, Zn electrodes were immersed in ethanol and distilled water to investigate the degradation characteristics of different solvents. Figure 8b shows a Zn electrode degraded by distilled water for 24 h. On the other hand, the Zn electrode was in a stable state in ethanol. As another representative study, in 2015, Hwang et al. measured EMG using biodegradable (poly(1,8-octanediol-co-citrate) (POC) as a substrate for an electrophysiology sensor [123]. The electrophysiology sensor was immersed in PBS solution (pH 10), to investigate its degradability (Figure 8c). As a result, the Mg electrode was degraded within a few hours, while SiO_2_ and POC were slowly dissolved within a few weeks. The dissolution kinetics of the proposed sensor depend on the temperature, pH, and ionic concentration of the solution, which have parametric effects on hydrolysis.

For strong adhesion to the skin, in 2022, Meng et al. manufactured a dry film electrode with polypyrrole (Ppy), acid-modified silk fibroin (AM-SF), and cellulose nanocrystals (CNC) [124]. They modified the tyrosine side chain of the SF protein with the sulfonic acid groups and obtained an interlocking structure between the positively-charged Ppy and negatively-charged SF networks. In the proposed film, Ppy acts as a CPM, calcium (Ca)-modified SF acts as an adhesive matrix, and CNC was used as a crosslinker, respectively. The degradation characteristic of Ppy@AM-SF/CNC film was investigated by immersing the film in PBS and protease XIV solutions (Figure 8d). Figure 8e shows a photograph of the degradable Ppy@AM-SF/CNC film in a Protease XIV condition at 37 °C. With the proposed conditions, the proposed film degraded into small fragments after 15 days through the catalytic proteolysis reaction of SF, owing to the Protease XIV enzyme.

Shao et al. demonstrated a hydrogel-based epidermal ECG sensor by combining cellulose nanofibers (CNF), polydopamine (PDA), and polyacrylamide (PAM) [125]. The proposed hydrogel has high ion-conductive, self-adhesive, and water-degradable characteristics. CNF, PDA, and PAM were cross-linked through hydrogen bonds between the functional groups of polymers. Additionally, ferric (Fe^3+^) interacted with polymers via metal chelation and was introduced to promote the degradation of the hydrogel through the Fenton-like reaction. During the degradation process, hydrophilic amide groups of the PAM chains absorb a large number of water molecules, which gradually decompose the hydrogen bonds between the polymer matrix chains. The penetrated oxygen molecules from the atmosphere slacken the network structure and oxidized the catechol groups of the tris-catechol-ferric complexes. Through the above steps, the ferric was released from the complexes and could participate in the Fenton-like reactions by reduction to Fe^2+^. The ammonium persulfate added to the gel synthesis process was activated by the released Fe^2+^. The Fenton-like reaction was promoted through the production of oxidant SO^4−^ radical, and the generated radicals consequently reacted with hydroxyl ions in the solution. The above reaction produces hydroxyl radical, which has a high reactivity, and decomposes the remaining gel components into small organic molecules. To investigate the changes in molecular weight of hydrogels quantitatively, gel permeation chromatography (GPC) was performed, and the molecular weight was reduced by half during the 30-day degradation test period.

In Section 3, we systematically reviewed biodegradable polymeric composite electrodes, with various materials and degradation mechanisms. The detail bio-signal sensing performance parameters of the proposed composites are summarized in Table 1 with a comparison with a commercial Ag/AgCl gel electrode.

## 4. Future Perspectives and Challenges

The research results presented above prove that biodegradable polymer electrodes have the potential to be used as a key material in various medical fields in the future. For instance, since biocompatible polymer composites are non-toxic to the human body, they can be utilized for long-term bio-signal collection, and this technology is expected to greatly contribute to maintaining life-expectancies through continuous health monitoring. Preventative, predictive, and personalized management of diseases can be achieved through electrophysiological sensing systems being integrated with IoT technologies. Furthermore, the biodegradable characteristics of polymer composite electrodes could prevent environmental pollution by reducing e-waste. However, the following technical barriers should be overcome for further development of the technology of biodegradable polymer electrophysiological sensors.

(1) One of the applications of bio-signal sensing electrodes is collecting electrophysiological signals over long-term periods by attaching them to the human body. The currently commercialized system has difficulty in continuous monitoring, due to its weight and bulky system size, which restricts the free movement of the patient when it is attached to the body. To overcome the aforementioned issue, it is essential to reduce the weight and size of the measurement system. In terms of application in an integrated sensing system, bio-signal sensing electrodes should be arranged with other components. For this integration, a high-level patterning process is required, and the combinations of materials and processes needs to be further diversified.
(2) The experimental conditions for biodegradation tests should be standardized. Most of biodegradable properties were investigated using a single specific solvent. To realize the full commercialization of biodegradable polymers, it is necessary to specifically characterize the biodegradable properties under various conditions, such as ambient humidity, solvent temperature, and acidity.
(3) The biocompatibility of by-products generated during decomposition should be considered. The organic material itself used as an electrode has biocompatibility when in the form of a polymer, but by-products occurring in the decomposition reaction may be harmful. Therefore, to utilize biodegradable electrodes for practical applications, a sensor design that guarantees the non-toxicity of by-products is required.
(4) The mechanical and chemical stability of polymer composites should be further improved. To continuously monitor electrophysiological signals with polymer composite electrodes, it is necessary to attach an epidermal sensor to the human body under daily. Therefore, composite film electrodes should guarantee sufficient resistance to variables that can occur in the conditions of daily life, such as sweat, movement, and pressure from external contact. For the practical utilization of electrodes, properties such as mechanical durability and adhesion of composites need to be further developed.
(5) Controllable degradation kinetics of components are required for specific applications, such as implantable devices. In the above-mentioned approaches, the degradation rate needs to be programmed for the desired conditions. To meet the above requirements, the control of the dissolution or degradation behavior of BFMs and CPMs needs to be improved.

**Figure 9:**
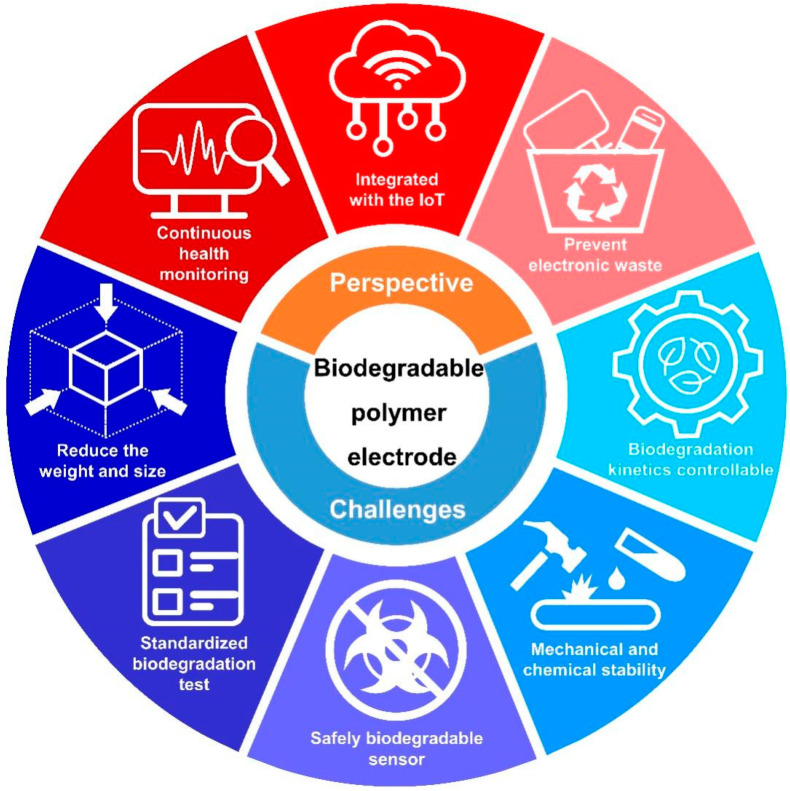
Overall schematic description of future perspectives and challenges for biodegradable polymer electrodes.

The future perspectives and challenges of the biodegradable polymer electrodes are summarized and illustrated with Figure 9.

## 5. Conclusions and Outlooks

In this review, we summarized functional organic materials for biodegradable polymeric epidermal electrodes into conductive, frame, and adhesive categories. The specific characteristics of electrophysiological signals, including ECG, EMG, EEG, and previous attempts to collect them using biodegradable electrodes were reviewed, with detailed degradation mechanisms. As the revisited studies suggest, the feasibility of biodegradable polymers for versatile uses in various electrophysiological sensing devices is promising. Various polymer composites for both wet and dry type electrodes potentially satisfy conductive and functional electrode roles, as well as having high compatibility with the skin and body. The gel-type electrodes introduced in this review have advantages in terms of their ease of fabrication, high ionic conductivity, and robust mechanical properties. However, as mentioned in the introduction, the performance of gel-type electrodes is affected by environmental conditions. From this point of view, dry electrodes may be an ideal skin-contact electrode, but there are many obstacles to the implementation of dry film electrodes with excellent adhesion, mechanical stability, and conductivity. Owing to the aforementioned issues, studies on polymer composites to improve gel-type electrodes have been previously attempted. Despite the challenges, there have been many recent attempts to implement ideal dry-type epidermal electrodes with robust mechano-electrical characteristics. We believe that such comprehensive analyses and the various attempts will open up an opportunity for valuable uses in the next generation of wearable or implantable healthcare devices.

## Figures and Tables

**Table 1:** Comparison of sensing properties according to fabrication technique with commercial Ag/AgCl electrodes.

Materials	Electrophysiology Signal	Signal-to-Noise Ratio	Impedances	Sensitivity	Fabrication Technique	Ref.
Ag/AgCl	ECG	N/A	6.2 kΩ at 1 MHz	N/A	N/A	[99]
Silk adhesive	ECG	N/A	1.5 kΩ at 1 MHz	N/A	Solution synthesis	[99]
Ag/AgCl	ECG, EMG	0.8159 dB	N/A	N/A	N/A	[100]
MXene-PAA-ACC hydrogel-based electrodes	ECG, EMG	19.96 dB	N/A	N/A	Solution synthesis	[100]
Ag/AgCl	ECG, EMG	32 dB	~37 kΩ at 100 Hz	N/A	N/A	[108]
Pencil-drawn electrophysiological electrodes	ECG, EMG	30 dB	~40 kΩ at 100 Hz	N/A	Sketch on office paper with 9B pencil	[108]
Ag/AgCl	ECG, EMG, EOG	32.2 dB	~50 kΩ at 10 Hz	N/A	N/A	[109]
Y-shaped kirigami structure electrode	ECG, EMG, EOG	22.8 dB	~70 kΩ at 10 Hz	N/A	Spin coating Laser cutting	[109]
Ag/AgCl	ECG, EMG	N/A	N/A	0.29	N/A	[124]
Ppy@AM-SF/CNC electrodes	ECG, EMG	N/A	N/A	0.45	Solution blended	[124]

## Data Availability

Not applicable.

## References

[1] Khairuddin A., Azir K.F.K., Kan P.E. Limitations and future of electrocardiography devices: A review and the perspective from the Internet of Things. Proceedings of the 2017 International Conference on Research and Innovation in Information Systems (ICRIIS).

[2] Rosli K.A., Omar M.H., Hasan A.F., Musa K.S., Fadzil M.F.M., Neu S.H. Development of electrocardiograph monitoring system. Proceedings of the MATEC Web of Conferences.

[3] Soroudi A., Hernández N., Berglin L., Nierstrasz V. (2019). Electrode placement in electrocardiography smart garments: A review. J. Electrocardiol..

[4] Tankisi H., Burke D., Cui L., de Carvalho M., Kuwabara S., Nandedkar S.D., Rutkove S., Stålberg E., van Putten M.J., Fuglsang-Frederiksen A. (2020). Standards of instrumentation of EMG. Clin. Neurophysiol..

[5] Simão M., Mendes N., Gibaru O., Neto P. (2019). A review on electromyography decoding and pattern recognition for human-machine interaction. IEEE Access.

[6] Liu S.-H., Lin C.-B., Chen Y., Chen W., Huang T.-S., Hsu C.-Y. (2019). An EMG patch for the real-time monitoring of muscle-fatigue conditions during exercise. Sensors.

[7] Teplan M. (2002). Fundamentals of EEG measurement. Meas. Sci. Rev..

[8] Alotaiby T., Abd El-Samie F.E., Alshebeili S.A., Ahmad I. (2015). A review of channel selection algorithms for EEG signal processing. EURASIP J. Adv. Signal Process.

[9] Kennett R. (2012). Modern electroencephalography. J. Neurol..

[10] Usakli A.B., Gurkan S., Aloise F., Vecchiato G., Babiloni F. (2010). On the use of electrooculogram for efficient human computer interfaces. Comput. Intell. Neurosci..

[11] Lopez A., Ferrero F.J., Valledor M., Campo J.C., Postolache O. A study on electrode placement in EOG systems for medical applications. Proceedings of the 2016 IEEE International Symposium on Medical Measurements and Applications (MeMeA).

[12] Hadjem M., Salem O., Naït-Abdesselam F. An ECG monitoring system for prediction of cardiac anomalies using WBAN. Proceedings of the 2014 IEEE 16th International conference on e-Health networking, Applications and Services (Healthcom).

[13] Askari S., Zhang M., Won D.S. An EMG-based system for continuous monitoring of clinical efficacy of Parkinson’s disease treatments. Proceedings of the 2010 Annual International Conference of the IEEE Engineering in Medicine and Biology.

[14] Antony A.R., Haneef Z. (2020). Systematic review of EEG findings in 617 patients diagnosed with COVID-19. Seizure.

[15] Romero L., Pueyo E., Fink M., Rodríguez B. (2009). Impact of ionic current variability on human ventricular cellular electrophysiology. Am. J. Physiol. Heart Circ. Physiol..

[16] O’Mahony C., Grygoryev K., Ciarlone A., Giannoni G., Kenthao A., Galvin P. (2016). Design, fabrication and skin-electrode contact analysis of polymer microneedle-based ECG electrodes. J. Micromech. Microeng..

[17] Kimura M., Nakatani S., Nishida S.-I., Taketoshi D., Araki N. (2020). 3D printable dry EEG electrodes with coiled-spring prongs. Sensors.

[18] Pan X., Wang Q., He P., Liu K., Ni Y., Ouyang X., Chen L., Huang L., Wang H., Tan Y. (2019). Mussel-inspired nanocomposite hydrogel-based electrodes with reusable and injectable properties for human electrophysiological signals detection. ACS Sustain. Chem. Eng..

[19] Luo J., Xing Y., Sun C., Fan L., Shi H., Zhang Q., Li Y., Hou C., Wang H. (2022). A bio-adhesive ion-conducting organohydrogel as a high-performance non-invasive interface for bioelectronics. Chem. Eng. J..

[20] Liu X., Chen X., Chi X., Feng Z., Yang C., Gao R., Li S., Zhang C., Chen X., Huang P. (2022). Biomimetic integration of tough polymer elastomer with conductive hydrogel for highly stretchable, flexible electronic. Nano Energy.

[21] Leleux P., Badier J.M., Rivnay J., Bénar C., Hervé T., Chauvel P., Malliaras G.G. (2014). Conducting polymer electrodes for electroencephalography. Adv. Healthc. Mater..

[22] Zahed M.A., Das P.S., Maharjan P., Barman S.C., Sharifuzzaman M., Yoon S.H., Park J.Y. (2020). Flexible and robust dry electrodes based on electroconductive polymer spray-coated 3D porous graphene for long-term electrocardiogram signal monitoring system. Carbon.

[23] An X., Stylios G.K. (2018). A hybrid textile electrode for electrocardiogram (ECG) measurement and motion tracking. Materials.

[24] Polk B.J., Stelzenmuller A., Mijares G., MacCrehan W., Gaitan M. (2006). Ag/AgCl microelectrodes with improved stability for microfluidics. Sens. Actuators B Chem..

[25] Huynh T.M., Nguyen T.S.V., Doan T.C.D., Dang C.M. (2019). Fabrication of thin film Ag/AgCl reference electrode by electron beam evaporation method for potential measurements. Adv. Nat. Sci. Nanosci. Nanotechnol..

[26] Das P.S., Yoon H.S., Kim J., Kim D.H., Park J.Y. (2018). Simple fabrication method of an ultrasensitive gold micro-structured dry skin sensor for biopotential recording. Microelectron. Eng..

[27] Kim C.H., Lee D.H., Youn J., Lee H., Jeong J. (2021). Simple and cost-effective microfabrication of flexible and stretchable electronics for wearable multi-functional electrophysiological monitoring. Sci. Rep..

[28] Jung H.-C., Moon J.-H., Baek D.-H., Lee J.-H., Choi Y.-Y., Hong J.-S., Lee S.-H. (2012). CNT/PDMS composite flexible dry electrodesfor long-term ECG monitoring. IEEE Trans. Biomed. Eng..

[29] Wang Y., Zhong X., Wang W., Yu D. (2021). Flexible cellulose/polyvinyl alcohol/PEDOT: PSS electrodes for ECG monitoring. Cellulose.

[30] Chiong J.A., Tran H., Lin Y., Zheng Y., Bao Z. (2021). Integrating Emerging Polymer Chemistries for the Advancement of Recyclable, Biodegradable, and Biocompatible Electronics. Adv. Sci..

[31] Lee S.M., Byeon H.J., Lee J.H., Baek D.H., Lee K.H., Hong J.S., Lee S.-H. (2014). Self-adhesive epidermal carbon nanotube electronics for tether-free long-term continuous recording of biosignals. Sci. Rep..

[32] Yu W., Jiang G., Liu D., Li L., Chen H., Liu Y., Huang Q., Tong Z., Yao J., Kong X. (2017). Fabrication of biodegradable composite microneedles based on calcium sulfate and gelatin for transdermal delivery of insulin. Mater. Sci. Eng. C.

[33] Zhang Y., Tao T.H. Transient Epidermal Electronics for Learning the Physiological Signatures. Proceedings of the 2020 IEEE 33rd International Conference on Micro Electro Mechanical Systems (MEMS).

[34] Bihar E., Corzo D., Hidalgo T.C., Rosas-Villalva D., Salama K.N., Inal S., Baran D. (2020). Fully inkjet-printed, ultrathin and conformable organic photovoltaics as power source based on cross-linked PEDOT: PSS electrodes. Adv. Mater. Technol..

[35] Hu X., Meng X., Zhang L., Zhang Y., Cai Z., Huang Z., Su M., Wang Y., Li M., Li F. (2019). A mechanically robust conducting polymer network electrode for efficient flexible perovskite solar cells. Joule.

[36] Xu H., Zhao X., Yang G., Ji X., Zhang X., Li L., Wu B., Ouyang X., Ni Y., Chen L. (2022). Modification of PEDOT: PSS towards high-efficiency OLED electrode via synergistic effect of carboxy and phenol groups from biomass derivatives. Chem. Eng. J..

[37] Wang T., Jing L.-C., Zhu Q., Ethiraj A.S., Tian Y., Zhao H., Yuan X.-T., Wen J.-G., Li L.-K., Geng H.-Z. (2020). Fabrication of architectural structured polydopamine-functionalized reduced graphene oxide/carbon nanotube/PEDOT: PSS nanocomposites as flexible transparent electrodes for OLEDs. Appl. Surf. Sci..

[38] Wustoni S., Hidalgo T.C., Hama A., Ohayon D., Savva A., Wei N., Wehbe N., Inal S. (2020). In Situ Electrochemical Synthesis of a Conducting Polymer Composite for Multimetabolite Sensing. Adv. Mater. Technol..

[39] Kim J.H., Kim S.M., Kim G., Yoon M.H. (2020). Designing Polymeric Mixed Conductors and Their Application to Electrochemical-Transistor-Based Biosensors. Macromol. Biosci..

[40] Li M., Nykypanchuk D., Cotlet M. (2018). Improving the responsivity of hybrid graphene–conductive polymer photodetectors via nanowire self-assembly. ACS Photonics.

[41] Pasupuleti K.S., Reddeppa M., Park B.-G., Peta K.R., Oh J.-E., Kim S.-G., Kim M.-D. (2020). Ag nanowire-plasmonic-assisted charge separation in hybrid heterojunctions of Ppy-PEDOT: PSS/GaN nanorods for enhanced UV photodetection. ACS Appl. Mater. Interfaces.

[42] Puthirath A.B., Baburaj A., Kato K., Salpekar D., Chakingal N., Cao Y., Babu G., Ajayan P.M. (2019). High sulfur content multifunctional conducting polymer composite electrodes for stable Li-S battery. Electrochim. Acta.

[43] Liu B., Bo R., Taheri M., Di Bernardo I., Motta N., Chen H., Tsuzuki T., Yu G., Tricoli A. (2019). Metal–organic frameworks/conducting polymer hydrogel integrated three-dimensional free-standing monoliths as ultrahigh loading Li–S battery electrodes. Nano Lett..

[44] Shirakawa H. (2001). Nobel lecture: The discovery of polyacetylene film—the dawning of an era of conducting polymers. Rev. Mod. Phys..

[45] Pang A.L., Arsad A., Ahmadipour M. (2021). Synthesis and factor affecting on the conductivity of polypyrrole: A short review. Polym. Adv. Technol..

[46] Jaymand M., Hatamzadeh M., Omidi Y. (2015). Modification of polythiophene by the incorporation of processable polymeric chains: Recent progress in synthesis and applications. Prog. Polym. Sci..

[47] Nederstedt H., Jannasch P. (2020). Synthesis, Phase Structure, and Ion Conductivity of Poly (p-phenylene) Functionalized with Lithium Trifluoromethanesulfonimide and Tetra (ethylene Oxide) Side Chains. ACS Appl. Energy Mater..

[48] Kayser L.V., Lipomi D.J. (2019). Stretchable conductive polymers and composites based on PEDOT and PEDOT: PSS. Adv. Mater..

[49] Zhang C., Kong X., Liu W., Yang J., Zhao H. (2021). Regulation of PANI nanofiber conductivity and its influence on the DC dielectric properties of LDPE. Polym. Test.

[50] Tajik S., Beitollahi H., Nejad F.G., Shoaie I.S., Khalilzadeh M.A., Asl M.S., Van Le Q., Zhang K., Jang H.W., Shokouhimehr M. (2020). Recent developments in conducting polymers: Applications for electrochemistry. RSC Adv..

[51] Magu T., Agobi A., Hitler L., Dass P. (2019). A review on conducting polymers-based composites for energy storage application. J. Chem. Rev..

[52] Namsheer K., Rout C.S. (2021). Conducting polymers: A comprehensive review on recent advances in synthesis, properties and applications. RSC Adv..

[53] Hosseini E.S., Dervin S., Ganguly P., Dahiya R. (2020). Biodegradable materials for sustainable health monitoring devices. ACS Appl. Bio Mater..

[54] Zinge C., Kandasubramanian B. (2020). Nanocellulose based biodegradable polymers. Eur. Polym. J..

[55] Priyadarshi R., Rhim J.-W. (2020). Chitosan-based biodegradable functional films for food packaging applications. Innov. Food Sci. Emerg. Technol..

[56] Salama H.E., Abdel Aziz M.S., Sabaa M.W. (2018). Novel biodegradable and antibacterial edible films based on alginate and chitosan biguanidine hydrochloride. Int. J. Biol. Macromol..

[57] Umuhoza D., Yang F., Long D., Hao Z., Dai J., Zhao A. (2020). Strategies for Tuning the Biodegradation of Silk Fibroin-Based Materials for Tissue Engineering Applications. ACS Biomater. Sci. Eng..

[58] Olaiya N.G., Surya I., Oke P.K., Rizal S., Sadiku E.R., Ray S.S., Farayibi P.K., Hossain M.S., Abdul Khalil H.P.S. (2019). Properties and Characterization of a PLA–Chitin–Starch Biodegradable Polymer Composite. Polymers.

[59] Kim H., Choi S., Hong Y., Chung J., Choi J., Choi W.-K., Park I.W., Park S.H., Park H., Chung W.-J. (2021). Biocompatible and biodegradable triboelectric nanogenerators based on hyaluronic acid hydrogel film. Appl. Mater. Today.

[60] Zong H., Wang B., Li G., Yan S., Zhang K., Shou Y., Yin J. (2020). Biodegradable High-Strength Hydrogels with Injectable Performance Based on Poly(l-Glutamic Acid) and Gellan Gum. ACS Biomater. Sci. Eng..

[61] Toledano M., Asady S., Toledano-Osorio M., García-Godoy F., Serrera-Figallo M.-A., Benítez-García J.A., Osorio R. (2020). Differential Biodegradation Kinetics of Collagen Membranes for Bone Regeneration. Polymers.

[62] Puppi D., Chiellini F. (2020). Biodegradable Polymers for Biomedical Additive Manufacturing. Appl. Mater. Today.

[63] Li C., Guo C., Fitzpatrick V., Ibrahim A., Zwierstra M.J., Hanna P., Lechtig A., Nazarian A., Lin S.J., Kaplan D.L. (2020). Design of biodegradable, implantable devices towards clinical translation. Nat. Rev. Mater..

[64] Shi J., Zhang L., Xiao P., Huang Y., Chen P., Wang X., Gu J., Zhang J., Chen T. (2018). Biodegradable PLA Nonwoven Fabric with Controllable Wettability for Efficient Water Purification and Photocatalysis Degradation. ACS Sustain. Chem. Eng..

[65] O’Donnell K.L., Oporto-Velásquez G.S., Comolli N. (2020). Evaluation of Acetaminophen Release from Biodegradable Poly (Vinyl Alcohol) (PVA) and Nanocellulose Films Using a Multiphase Release Mechanism. Nanomaterials.

[66] Peng X., Dong K., Ye C., Jiang Y., Zhai S., Cheng R., Liu D., Gao X., Wang J., Wang Z.L. (2020). A breathable, biodegradable, antibacterial, and self-powered electronic skin based on all-nanofiber triboelectric nanogenerators. Sci. Adv..

[67] Liu Y., Zhu G., Yang H., Wang C., Zhang P., Han G. (2018). Bending behaviors of fully covered biodegradable polydioxanone biliary stent for human body by finite element method. J. Mech. Behav. Biomed. Mater..

[68] Lyu J.S., Lee J.-S., Han J. (2019). Development of a biodegradable polycaprolactone film incorporated with an antimicrobial agent via an extrusion process. Sci. Rep..

[69] Urbánek T., Jäger E., Jäger A., Hrubý M. (2019). Selectively Biodegradable Polyesters: Nature-Inspired Construction Materials for Future Biomedical Applications. Polymers.

[70] Joshi D.C., Saxena S., Jayakannan M. (2019). Development of l-Lysine Based Biodegradable Polyurethanes and Their Dual-Responsive Amphiphilic Nanocarriers for Drug Delivery to Cancer Cells. ACS Appl. Polym. Mater..

[71] Yuen Y.A., Porcarelli L.H., Aguirresarobe R., Sanchez-Sanchez A., Del Agua I., Ismailov U.G., Malliaras G., Mecerreyes D., Ismailova E., Sardon H. (2018). Biodegradable Polycarbonate Iongels for Electrophysiology Measurements. Polymers.

[72] Cristofaro F., Gigli M., Bloise N., Chen H., Bruni G., Munari A., Moroni L., Lotti N., Visai L. (2018). Influence of the nanofiber chemistry and orientation of biodegradable poly(butylene succinate)-based scaffolds on osteoblast differentiation for bone tissue regeneration. Nanoscale.

[73] Huang Y., Du Z., Wei P., Chen F., Guan B., Zhao Z., Zhang X., Cai Q., Mao J., Leng H. (2020). Biodegradable microspheres made of conductive polyorganophosphazene showing antioxidant capacity for improved bone regeneration. Chem. Eng. J..

[74] Niewolik D., Bednarczyk-Cwynar B., Ruszkowski P., Kazek-Kęsik A., Dzido G., Jaszcz K. (2022). Biodegradable and Bioactive Carriers Based on Poly(betulin disuccinate-co-sebacic Acid) for Rifampicin Delivery. Pharmaceutics.

[75] Sanchez-Salvador J.L., Balea A., Monte M.C., Negro C., Blanco A. (2021). Chitosan grafted/cross-linked with biodegradable polymers: A review. Int. J. Biol. Macromol..

[76] Wang W., Liu S., Chen B., Yan X., Li S., Ma X., Yu X. (2019). DNA-Inspired Adhesive Hydrogels Based on the Biodegradable Polyphosphoesters Tackified by a Nucleobase. Biomacromolecules.

[77] Ahmad A.F., Aziz S.A., Obaiys S.J., Zaid M.H.M., Matori K.A., Samikannu K., Aliyu U.S.A. (2020). Biodegradable Poly (lactic acid)/Poly (ethylene glycol) Reinforced Multi-Walled Carbon Nanotube Nanocomposite Fabrication, Characterization, Properties, and Applications. Polymers.

[78] Peng X., Dong K., Wu Z., Wang J., Wang Z.L. (2021). A review on emerging biodegradable polymers for environmentally benign transient electronic skins. J. Mater. Sci..

[79] Donato R.K., Mija A. (2019). Keratin associations with synthetic, biosynthetic and natural polymers: An extensive review. Polymers.

[80] Wang B., Yang W., McKittrick J., Meyers M.A. (2016). Keratin: Structure, mechanical properties, occurrence in biological organisms, and efforts at bioinspiration. Prog. Mater. Sci..

[81] Zhou D., Li S., Pei M., Yang H., Gu S., Tao Y., Ye D., Zhou Y., Xu W., Xiao P. (2020). Dopamine-modified hyaluronic acid hydrogel adhesives with fast-forming and high tissue adhesion. ACS Appl. Mater. Interfaces.

[82] Correia C., Sousa R.O., Vale A.C., Peixoto D., Silva T.H., Reis R.L., Pashkuleva I., Alves N.M. (2022). Adhesive and biodegradable membranes made of sustainable catechol-functionalized marine collagen and chitosan. Colloids Surf. B Biointerfaces.

[83] Ortiz-Fernández A., Ríos-Soberanis C.R., Chim-Chi Y.A., Moo-Huchin V.M., Estrada-León R.J., Pérez-Pacheco E. (2021). Optimization of biodegradable starch adhesives using response surface methodology. Polym. Bull..

[84] Hou F., Jiang W., Zhang Y., Tang J., Li D., Zhao B., Wang L., Gu Y., Cui W., Chen L. (2022). Biodegradable dual-crosslinked adhesive glue for fixation and promotion of osteogenesis. Chem. Eng. J..

[85] Pandey N., Hakamivala A., Xu C., Hariharan P., Radionov B., Huang Z., Liao J., Tang L., Zimmern P., Nguyen K.T. (2018). Biodegradable nanoparticles enhanced adhesiveness of mussel-like hydrogels at tissue interface. Adv. Healthc. Mater..

[86] Zhang W., Wang R., Sun Z., Zhu X., Zhao Q., Zhang T., Cholewinski A., Yang F.K., Zhao B., Pinnaratip R. (2020). Catechol-functionalized hydrogels: Biomimetic design, adhesion mechanism, and biomedical applications. Chem. Soc. Rev..

[87] Rahimnejad M., Zhong W. (2017). Mussel-inspired hydrogel tissue adhesives for wound closure. RSC Adv..

[88] Jung H., Kim M.K., Lee J.Y., Choi S.W., Kim J. (2020). Adhesive hydrogel patch with enhanced strength and adhesiveness to skin for transdermal drug delivery. Adv. Funct. Mater..

[89] Barry D.T., Gordon K.E., Hinton G.G. (1990). Acoustic and surface EMG diagnosis of pediatric muscle disease. Muscle Nerve.

[90] Kullmann F., Hollerbach S., Lock G., Holstege A., Dierks T., Schölmerich J. (2001). Brain electrical activity mapping of EEG for the diagnosis of (sub) clinical hepatic encephalopathy in chronic liver disease. Eur. J. Gastroenterol. Hepatol..

[91] Vijayavanan M., Rathikarani V., Dhanalakshmi P. (2014). Automatic classification of ECG signal for heart disease diagnosis using morphological features. Int. J. Comput. Sci. Eng..

[92] Tayeb Z., Bose R., Dragomir A., Osborn L.E., Thakor N.V., Cheng G. (2020). Decoding of pain perception using EEG Signals for a Real-Time Reflex System in prostheses: A case Study. Sci. Rep..

[93] Rho G., Callara A.L., Condino S., Ghiasi S., Nardelli M., Carbone M., Ferrari V., Greco A., Scilingo E.P. A preliminary quantitative EEG study on Augmented Reality Guidance of Manual Tasks. Proceedings of the 2020 IEEE International Symposium on Medical Measurements and Applications (MeMeA).

[94] Awasthi A.K., Cucchiella F., D’Adamo I., Li J., Rosa P., Terzi S., Wei G., Zeng X. (2018). Modelling the correlations of e-waste quantity with economic increase. Sci. Total Environ..

[95] Liu C.-M., Chang S.-L., Yeh Y.-H., Chung F.-P., Hu Y.-F., Chou C.-C., Hung K.-C., Chang P.-C., Liao J.-N., Chan Y.-H. (2021). Enhanced detection of cardiac arrhythmias utilizing 14-day continuous ECG patch monitoring. Int. J. Cardiol..

[96] Baloglu U.B., Talo M., Yildirim O., San Tan R., Acharya U.R. (2019). Classification of myocardial infarction with multi-lead ECG signals and deep CNN. Pattern Recognit. Lett..

[97] Kamishima K., Yamada Y., Kawarai H., Kudo K., Shimazaki K., Henmi R., Honda A., Gunji K., Uno M., Haruta S. (2013). A case of variant angina treated with a pacemaker for cardiopulmonary arrest due to complete atrioventricular block and pulseless electrical activity. J. Arrhythm..

[98] Hurst J.W. (1998). Naming of the waves in the ECG, with a brief account of their genesis. Circulation.

[99] Seo J.W., Kim H., Kim K., Choi S.Q., Lee H.J. (2018). Calcium-modified silk as a biocompatible and strong adhesive for epidermal electronics. Adv. Funct. Mater..

[100] Li X., He L., Li Y., Chao M., Li M., Wan P., Zhang L. (2021). Healable, degradable, and conductive MXene nanocomposite hydrogel for multifunctional epidermal sensors. ACS Nano.

[101] McGill K.C., Dorfman L.J. (1985). Automatic decomposition electromyography (ADEMG): Validation and normative data in brachial biceps. Electroencephalogr. Clin. Neurophysiol..

[102] De Luca C.J., Adam A., Wotiz R., Gilmore L.D., Nawab S.H. (2006). Decomposition of surface EMG signals. J. Neurophysiol..

[103] Mahbub Z.B., Rabbani K. (2007). Frequency domain analysis to identify neurological disorders from evoked EMG responses. J. Biol. Phys..

[104] Esposito F., Veicsteinas A., Orizio C., Malgrati D. (1996). Time and frequency domain analysis of electromyogram and sound myogram in the elderly. Eur. J. Appl. Physiol. Occup. Physiol..

[105] Phinyomark A., Chujit G., Phukpattaranont P., Limsakul C., Hu H. A preliminary study assessing time-domain EMG features of classifying exercises in preventing falls in the elderly. Proceedings of the 2012 9th International Conference on Electrical Engineering/Electronics, Computer, Telecommunications and Information Technology.

[106] Mete T., Aydin Y., Saka M., Cinar Yavuz H., Bilen S., Yalcin Y., Arli B., Berker D., Guler S. (2013). Comparison of efficiencies of michigan neuropathy screening instrument, neurothesiometer, and electromyography for diagnosis of diabetic neuropathy. Int. J. Endocrinol..

[107] De Visser B.O., Goor C. (1974). Electromyographic and reflex study in idiopathic and symptomatic trigeminal neuralgias: Latency of the jaw and blink reflexes. J. Neurol. Neurosurg. Psychiatry.

[108] Xu Y., Zhao G., Zhu L., Fei Q., Zhang Z., Chen Z., An F., Chen Y., Ling Y., Guo P. (2020). Pencil–paper on-skin electronics. Proc. Natl. Acad. Sci. USA.

[109] Won Y., Lee J.J., Shin J., Lee M., Kim S., Gandla S. (2021). Biocompatible, Transparent, and High-Areal-Coverage Kirigami PEDOT: PSS Electrodes for Electrooculography-Derived Human–Machine Interactions. ACS Sens..

[110] Zhang Y., Tao T.H. (2019). Skin-friendly electronics for acquiring human physiological signatures. Adv. Mater..

[111] Kulkarni N.N., Bairagi V. Electroencephalogram based diagnosis of Alzheimer disease. Proceedings of the 2015 IEEE 9th International Conference on Intelligent Systems and Control (ISCO).

[112] Seeck M., Koessler L., Bast T., Leijten F., Michel C., Baumgartner C., He B., Beniczky S. (2017). The standardized EEG electrode array of the IFCN. Clin. Neurophysiol..

[113] Oon H.N., Saidatul A., Ibrahim Z. Analysis on Non-linear features of electroencephalogram (EEG) signal for neuromarketing application. Proceedings of the 2018 International Conference on Computational Approach in Smart Systems Design and Applications (ICASSDA).

[114] Chatterjee R., Datta A., Sanyal D.K. (2019). Ensemble learning approach to motor imagery EEG signal classification. Machine Learning in Bio-Signal Analysis and Diagnostic Imaging.

[115] Nandi R., Agam Y., Amdursky N. (2021). A Protein-Based Free-Standing Proton-Conducting Transparent Elastomer for Large-Scale Sensing Applications. Adv. Mater..

[116] Yu K.J., Kuzum D., Hwang S.-W., Kim B.H., Juul H., Kim N.H., Won S.M., Chiang K., Trumpis M., Richardson A.G. (2016). Bioresorbable silicon electronics for transient spatiotemporal mapping of electrical activity from the cerebral cortex. Nat. Mater..

[117] Manouchehri S., Bagheri B., Rad S.H., Nezhad M.N., Kim Y.C., Park O.O., Farokhi M., Jouyandeh M., Ganjali M.R., Yazdi M.K. (2019). Electroactive bio-epoxy incorporated chitosan-oligoaniline as an advanced hydrogel coating for neural interfaces. Prog. Org..

[118] Lee Y., Yim S.-G., Lee G.W., Kim S., Kim H.S., Hwang D.Y., An B.-S., Lee J.H., Seo S., Yang S.Y. (2020). Self-adherent biodegradable gelatin-based hydrogel electrodes for electrocardiography monitoring. Sensors.

[119] Wan S., Wu N., Ye Y., Li S., Huang H., Chen L., Bi H., Sun L. (2021). Highly Stretchable Starch Hydrogel Wearable Patch for Electrooculographic Signal Detection and Human–Machine Interaction. Small Struct..

[120] Arquilla K., Webb A.K., Anderson A.P. (2020). Textile electrocardiogram (ECG) electrodes for wearable health monitoring. Sensors.

[121] bin Ahmad M.A.S., Harun F.K.C., Wicaksono D.H. Hybrid flexible circuit on cotton fabric for wearable electrocardiogram monitoring. Proceedings of the 2017 International Electronics Symposium on Engineering Technology and Applications (IES-ETA).

[122] Yi N., Cheng Z., Yang L., Edelman G., Xue C., Ma Y., Zhu H., Cheng H. (2018). Fully water-soluble, high-performance transient sensors on a versatile galactomannan substrate derived from the endosperm. ACS Appl. Mater. Interfaces.

[123] Hwang S.-W., Lee C.H., Cheng H., Jeong J.-W., Kang S.-K., Kim J.-H., Shin J., Yang J., Liu Z., Ameer G.A. (2015). Biodegradable elastomers and silicon nanomembranes/nanoribbons for stretchable, transient electronics, and biosensors. Nano Lett..

[124] Meng L., Fu Q., Hao S., Xu F., Yang J. (2022). Self-adhesive, biodegradable silk-based dry electrodes for epidermal electrophysiological monitoring. Chem. Eng. J..

[125] Shao Z., Hu X., Cheng W., Zhao Y., Hou J., Wu M., Xue D., Wang Y. (2020). Degradable self-adhesive epidermal sensors prepared from conductive nanocomposite hydrogel. Nanoscale.

